# Early relational health training in Canadian paediatric residency programs: A national program director survey

**DOI:** 10.1093/pch/pxaf136

**Published:** 2026-01-23

**Authors:** Katherine M Matheson, Nicole Sheridan, Sophie Zarb, Sara Citron, Michelle Ward, Blair Hammond

**Affiliations:** Department of Psychiatry, Children's Hospital of Eastern Ontario, Ottawa, Ontario, Canada; Faculty of Medicine, University of Ottawa, Ottawa, Ontario, Canada; Mental Health Research, Children's Hospital of Eastern Ontario Research Institute, Ottawa, Ontario, Canada; Mental Health Research, Children's Hospital of Eastern Ontario Research Institute, Ottawa, Ontario, Canada; Faculty of Medicine, University of Ottawa, Ottawa, Ontario, Canada; Mental Health Research, Children's Hospital of Eastern Ontario Research Institute, Ottawa, Ontario, Canada; Pediatrics, Whitehorse General Hospital, Whitehorse, Yukon, Canada; Department of Psychiatry, Children's Hospital of Eastern Ontario, Ottawa, Ontario, Canada; Faculty of Medicine, University of Ottawa, Ottawa, Ontario, Canada; Mental Health Research, Children's Hospital of Eastern Ontario Research Institute, Ottawa, Ontario, Canada; Mount Sinai Parenting Center, Icahn School of Medicine at Mount Sinai, New York, USA; Department of Pediatrics, Icahn School of Medicine at Mount Sinai, 1184 5th Avenue, New York, USA

**Keywords:** Early relational health, Mental health, Paediatric residency training, Paediatric subspecialty, Medical education, Curriculum development

## Abstract

**Objectives:**

Early Relational Health (ERH)—a foundational determinant of lifelong mental and physical health—is emerging as a critical component of paediatric practice. However, its integration into Canadian paediatric residency training remains poorly defined. This study conducted an environmental scan of Canadian paediatric residency programs to examine the current state of ERH education, as reported by Program Directors (PDs).

**Methods:**

PDs from Canadian paediatric residency programs were invited to complete an anonymous survey (September–December 2023). The survey explored PDs' definitions of ERH, existing training opportunities, perceptions of how well ERH is addressed, and motivators for incorporating structured curricula. Data were analyzed using descriptive statistics.

**Results:**

Ten PDs completed the survey (37% response rate). All (100%) General Pediatrics PDs reported their program teaches positive parenting and early child development “Not Very Well,” compared with 20% of subspecialty PDs. Sixty per cent of General Pediatrics PDs and all Subspecialty PDs rated an ERH curriculum as “Very Important” for their learners. Nearly all respondents expressed interest in implementing a structured, evidence-based, self-guided ERH curriculum to improve resident knowledge and skills.

**Discussion:**

This is the first study to evaluate ERH training within Canadian paediatric residency programs. While some subspecialty programs have integrated ERH content, most General Pediatrics programs identified clear training gaps. All PDs recognized ERH as an essential topic despite limited formal education opportunities. Development of a structured, competency-based ERH curriculum represents a key next step in advancing paediatric training and care quality in Canada.

## INTRODUCTION

Secure early attachments between children and caregivers foster positive childhood experiences and promote optimal physical, cognitive, emotional, and social development (1,2). In contrast, adverse childhood experiences are associated with a wide array of long-term health challenges, including increased risk for mental illness and chronic physical conditions (3). Emerging evidence now highlights the protective impact of positive childhood experiences, which are linked to improved psychosocial outcomes and reduced risk of mental and physical health symptoms (4). Safe, stable, and nurturing relationships—particularly those within the parent-child dyad—can buffer the effects of adversity and enhance resilience (5–7).

Within this context, the term *Early Relational Health* (ERH) has been introduced to describe the clinical domain that centres on the emotional connections between young children and their caregivers as a critical component of health. According to the Canadian Paediatric Society (CPS) and the American Academy of Pediatrics (AAP), ERH supports resilience through nurturing relationships and is essential to healthy development, especially for children exposed to adversity (8). Both organizations have emphasized the need to integrate ERH into paediatric practice and medical education. Clinical encounters that address ERH are supported by dedicated time, strong clinician-caregiver partnerships, and an emphasis on both child development and family well-being (9).

Despite these developments, the degree to which ERH has been incorporated into paediatric residency training varies. In the United States, a national survey found that although most residency program leaders considered ERH education essential, only 11% felt it was being taught effectively (10). Previous studies of targeted ERH interventions, such as training in evidence-based parenting programs (e.g., Triple P, Circle of Security) and structured curricula like *Keystones of Development*, have shown improvements in both residents' competencies and caregiver outcomes (11,12). Additionally, educational resources (e.g., AAP's ERH case-based modules) have been developed to facilitate integration of ERH into training and practice (13). However, despite the resources available to increase programs' capacity for teaching ERH, gaps remain in paediatric training related to early childhood mental health (MH), particularly during infancy and toddlerhood (14–16).

The Royal College of Physicians and Surgeons of Canada's specialty training requirements outline necessary training in paediatrics residency. For example, child and youth MH and community paediatrics education must be included, but the structure and content of this training varies by program (17). Some programs may require residents to complete a dedicated developmental paediatrics or MH rotation during their core residency training, while other programs may teach their trainees this content through other experiences (e.g., longitudinal clinical experiences, didactic lectures, etc.). Prior studies indicate that residents feel underprepared in key areas such as developmental-behavioural and social paediatrics, and that they desire greater exposure (16,18). Despite a shared North American interest in advancing ERH education, Canadian engagement in prior studies has been limited—no Canadian programs participated in Martin et al.'s 2020 survey (10).

To address this gap, our study conducted an environmental scan of ERH training across Canadian paediatric residency and subspecialty programs. Our objective was to explore how ERH is currently taught, the extent of trainee exposure, and the perceived drivers and barriers to implementing ERH curricula as identified by Program Directors (PDs).

## METHODS

This study was approved by the CHEO Research Ethics Board (23/80X) and adhered to the Checklist of Reporting of Survey Studies (Supplementary Material S1) (19). PDs and Associate Program Directors from Canadian paediatric residency and subspecialty fellowship programs most relevant (specifically Developmental Pediatrics, Child Development, Community Pediatrics) accredited by the Royal College of Physicians and Surgeons of Canada were invited to participate in this study. A total of 27 programs were eligible for inclusion; 17 general paediatrics programs and 10 subspecialty programs.

PDs were recruited via email and asked to complete an anonymous, voluntary, self-report survey hosted on REDCap (20) between September and December 2023. Separate surveys were developed for General Pediatric Residency and Subspecialty Fellowship PDs (Supplementary Material S2). Respondents were asked to answer based on their specific program context (e.g., fellowship PDs completed the survey for their subspecialty, not for core residency rotations). The surveys were adapted from a similar U.S. study by Martin et al. (10). Each survey contained 28 questions exploring PD demographics, current ERH training opportunities, trainee exposure, and perceived resident competence in ERH topics. Participants also provided insights on barriers to implementing ERH curricula and the motivations for increasing ERH training in their programs. Quantitative data were analyzed using descriptive statistics in IBM SPSS (21).

## RESULTS

PDs from all 17 general paediatric residency programs and 10 subspecialty programs were invited to participate. Of the 27 eligible programs, 10 PDs responded (37% response rate). The respondents were evenly distributed between larger General Pediatrics programs (5/17; 29.4% response rate) and smaller Subspecialty programs (5/10; 50% response rate). A detailed breakdown of participant characteristics is provided in Table 1.

**Table 1.** Program director participant characteristics

Responses	General paediatrics	Paediatric subspecialty
	n (%)	
Position		
Developmental paediatrics subspecialist	0 (0)	4 (80)
Other subspecialist paediatrician	3 (60)	1 (20)
General paediatrician	2 (40)	
Resident/fellow clinic preceptor		
Yes	2 (40)	5 (100)
No	3 (60)	
Location		
Urban only	3 (60)	4 (80)
Urban, suburban and rural	2 (40)	1 (20)
Program size*		
Small	1 (20)	5 (100)
Medium	1 (20)	
Large	3 (60)	

^*^Respondants were provided different definitions for program size options for general paediatrics residency programs and subspecialty programs. General paediatric residency options were Small (<10 residents/year), Medium (10 to 30 residents/year), and Large (>30 residents/year). Paediatric subspecialty options were defined as Small (<5 residents/year), Medium (5 to 10 residents/year), and Large (>10 residents per year).

When asked to define ERH within their program, responses were mixed for both program types, with 2/5 General Pediatric PDs providing a specific age range and 3/5 for subspecialty PDs. Only 30% of respondents provided a specific definition, while 40% did not have a defined concept of ERH and 30% were unsure. In core residency, 40% of respondents reported that their residents receive between 2 and 4 hours of didactic ERH training during their residency, while 60% reported the same for their subspecialty programs. Notably, none of the programs offered e-learning modules on ERH. In terms of clinical exposure, responses varied greatly between program types. Four out of five subspecialty PDs (80%) indicated that residents are required to complete a rotation or experience that includes ERH training (e.g., Social or Developmental-Behavioural Pediatrics rotations). In contrast, only 20% of General Pediatric PDs reported the same requirement. However, of the five programs with dedicated ERH exposure, only one offered experience with evidence-based ERH interventions, such as the Triple P Parenting program or Parent-Child Interaction Therapy.

**Figure 1.**
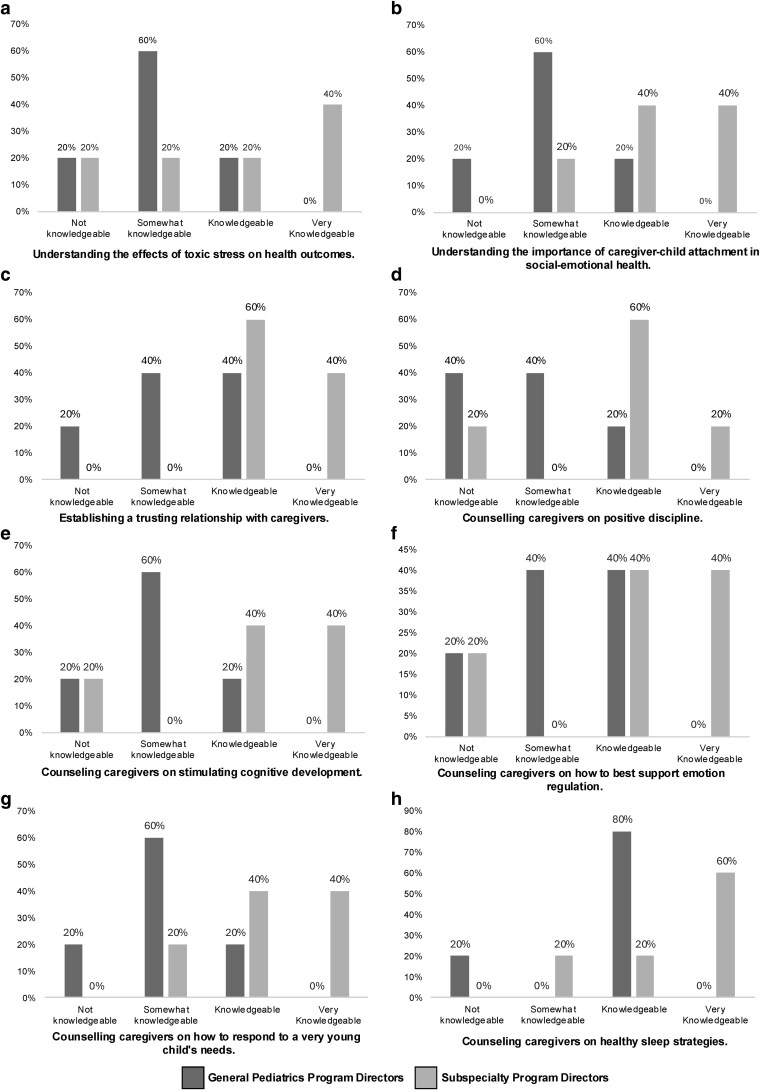
Perceived resident knowledge in early relational health.

Regarding residents' knowledge and competencies in ERH, PDs reported that trainees demonstrated at least some understanding across several domains. Eighty per cent of PDs rated their residents as “Somewhat Knowledgeable” or higher in counselling caregivers on sleep strategies and in establishing trusted relationships with caregivers. Among subspecialty programs, 80% of PDs rated their trainees as “Knowledgeable” or “Very Knowledgeable” in additional key areas, including counselling caregivers on responding to young children's needs, promoting cognitive development, understanding the effects of toxic stress on development, and recognising the role of caregiver–child attachment in social–emotional health (Figure 1). In contrast, 80% of General Pediatric PDs rated their trainees are being “Not Knowledgeable” or “Somewhat Knowledgeable” in these aforementioned categories.

When asked how well their programs teach parenting behaviours that promote early childhood development, all General Pediatric PDs reported teaching these topics “Not Very Well” (Table 2). The predominant reasons cited were the absence of a structured curriculum and a lack of faculty expertise (80%). When asked the same question to Subspecialty PDs, responses were rated more positively with 80% of respondents reporting “Moderately well” or “Very Well”.

**Table 2.** Current context of early relational health in paediatric programs

Responses	General paediatrics	Paediatric subspecialty
**Total hours of lectures/teaching sessions**		
Less than 2 hours	3 (60)	1 (20)
2 to 4 hours	2 (40)	3 (60)
More than 4 hours		1 (20)
**Types of teaching (multiple selections allowed)**		
Lecture	2 (40)	4 (80)
Small group discussions		3 (60)
Clinical teaching/rotation	1 (20)	1 (20)
Unsure	1 (20)	
No response	2 (40)	
**Recommended ERH reading list**		
Yes—Informal reading list (CPS statement/guideline)	0 (0)	3 (60)
No	5 (100)	2 (40)
Unsure		
**Mandatory ERH rotation/experience**		
No	4 (80)	1 (20)
Yes	1 (20)	4 (80)
**Availability of ERH learning module for residents**		
No	5 (100)	5 (100)
Yes	0 (0)	0 (0)
**How well program educates residents on EHR**		
Very well		1 (20)
Moderately well		3 (60)
Not very well	5 (100)	1 (20)
Reasons why:		
No/minimal curriculum available	4 (80)	
Lack of faculty experience	4 (80)	
Competing priorities	3 (60)	1
Cost	2 (40)	
Time	2 (40)	
Faculty buy-in	1 (20)	
Opportunities available	1 (20)	
**Importance of ERH curriculum in residency training**		
Very important	2 (40)	4 (80)
Important	1 (20)	1 (20)
Somewhat important	2 (40)	
Not important	0 (0)	
**Staff affiliated with program expressed interest in ERH**		
Yes	1 (20)	2 (40)
No	1 (20)	2 (4)
Unsure	3 (60)	
No response		1 (20)

### Preferences for an ERH curriculum

When asked the level of importance of learning ERH during paediatric training, all subspecialty PDs and 60% of General Pediatric PDs expressed “Important” or “Very Important”. PDs identified the following as the most prominent factors that would increase the likelihood of implementing EHR into their programs: the curriculum addresses program core competencies (80%), increases residents' knowledge and skills (50%) and can be woven into the existing program without displacing other topics (50%; Table 3). Additionally, almost all respondents preferred a curriculum that included self-guided modules with instruction on incorporating EHR into visits with young children and their caregivers (90%), along with learning resources on specific parenting topics (e.g., videos/handouts/tips on discipline, attachment, routines, sleep, etc.; 80%). Perceived barriers to implementing an ERH curriculum included competing priorities, no curriculum already available, lack of resident time, and a lack of faculty experience.

**Table 3.** Motivators and barriers for implementing an early relational health curriculum

Responses	General paediatrics	Paediatric subspecialty
**Motivators for implementing an ERH curriculum**		
Curriculum addresses the specific core competencies of your program	4 (80)	4 (80)
Curriculum can be woven into the existing program without any topics	4 (80)	1 (20)
Evidence that the curriculum increases residents' knowledge and skills	4 (80)	1 (2)
Evidence that this curriculum improves patient satisfaction	2 (40)	2 (40)
Evidence of residents' satisfaction with the curriculum	2 (40)	2 (40)
Evidence of quality of the caregiver-child relationship influences child outcomes	1 (20)	1 (20)
Positive feedback from previous residents who completed the curriculum	0 (0)	1 (20)
**Tools of interest to be included in a curriculum**		
Self-guided learning modules (no faculty facilitation required) that model weaving caregiver curriculum into visits with young children and their caregiver(s)	5 (100)	4 (80)
Learning resources for residents on specific parenting topics	4 (80)	4 (80)
Learning resources for caregivers on specific parenting topics	2 (40)	2 (40)
Parenting tips that can be emailed or texted to residents	2 (40)	2 (40)
PowerPoint slides for faculty lectures on specific parenting topics	2 (40)	3 (60)
Parenting tips that can be emailed or texted to caregivers	1 (20)	2 (40)
Live warmline support for residents to curriculum topics/parenting questions	0 (0)	1 (20)
**Barriers faced for implementation (n = 7)**		
More important priorities to implement	3 (60)	1 (33.3)
A lack of “buy-in” from attending physicians that this is a priority	3 (60)	0 (0)
Residents won’t have time to complete	2 (40)	1 (33.3)
A lack of faculty with the requisite knowledge base to implement, oversee, or answer questions	1 (20)	1 (33.3)
**Topics interested in being covered in curriculum**		
Promoting secure caregiver-child attachment	5 (100)	5 (100)
Tantrums	5 (100)	5 (100)
Positive discipline	4 (80)	4 (80)
Promoting emotion regulation	4 (80)	4 (80)
Promotion emotional literacy (e.g., communicating and expressing feelings)	4 (80)	4 (80)
Healthy sleep habits	3 (60)	4 (80)
Promoting independent exploration skills	2 (40)	4 (80)

## DISCUSSION

As ERH and positive childhood experiences become increasingly recognized as essential to childrens' overall health, the findings of this study underscore the variable and often insufficient integration of ERH training into Canadian General Pediatric core residency and subspecialty programs. Despite broad consensus among PDs about the importance of ERH within residency training, significant gaps persist in how effectively these concepts are taught and the extent to which trainees are exposed to relevant training opportunities.

Our results reveal that there are limited offerings of formal ERH training in core paediatric residency programs, but moderate levels of opportunities in specific subspecialty programs (Developmental Pediatrics, Child Development, Community Pediatrics). Core paediatric training sets the foundation for all paediatric trainees to go on to work effectively with children and families in their future, whether or not they complete subspecialty training in any domain afterwards. In this study, General Pediatric PDs recognized the importance of ERH into core training with nearly all PDs expressing strong support for integrating such training into their programs. PDs noted limited offerings for formal ERH training and over half of these PDs reported their residents are either not knowledgeable or only somewhat knowledgeable on ERH topics. These findings align with previous research showing that paediatric residents often feel inadequately prepared to address early childhood MH, particularly in domains such as attachment and toxic stress, which are crucial for managing paediatric MH across the lifespan (15,16,18,22).

Not surprisingly, more opportunities for training and higher level of knowledge are reported by subspecialty training programs that have a focus on this area, such as developmental paediatrics. Most subspecialty PDs expressed that their trainees were required to complete a rotation which incorporates ERH and more than half reporting that trainees were knowledgeable or very knowledgeable in almost all ERH topics when asked. Even so, PDs for these programs reported almost no training that includes evidence-based ERH interventions and these PDs also expressed strong support for the integration of ERH into their respective programs.

While most of the specific subspecialty programs surveyed do offer rotations or experiences incorporating ERH, these remain rare during core residency, and when offered, they often lack exposure to evidence-based interventions. This scarcity of formal training, combined with the absence of structured curricula and faculty expertise, highlights the need for targeted educational interventions to bridge these gaps. Interestingly, despite these challenges, nearly all PDs expressed strong support for the integration of ERH into their respective programs. Many PDs suggested that a self-directed, modular curriculum, with additional low-cost resources such as videos, handouts, and tip sheets, would be an effective strategy for enhancing ERH education. The overwhelming desire for such a curriculum at all levels of paediatric training reflects the increasing recognition of ERH's importance in clinical practice, underscoring its potential to foster better clinical outcomes for children and families.

These findings are particularly relevant when considering the broader context of paediatric training in North America. In the United States, several successful models for incorporating ERH training into paediatric residency curricula have been developed (10–12,14). These include curricula like *Keystones of Development* and evidence-based parenting programs, which have shown to improve both resident competency and patient outcomes (10,23). Our study suggests that Canadian programs can draw from these successful models and adapt them to local contexts, while also addressing identified barriers from study participants (e.g., lack of time and faculty experience).

This study represents the first environmental scan of ERH training in Canadian paediatric residency and subspecialty programs. This study is limited by its survey response rate, which mirrors the uptake of a similar study completed with American PDs (10). Nonetheless, the data provided valuable insights into current practices and gaps in the ERH training across a variety of clinical training experiences, highlighting that at least in some programs there is currently limited training in ERH. Of those who responded, however, there was strong recognition of the importance of ERH education.

The CPS recommends more focused ERH training for all medical trainees. However, this study shows that even in training programs specifically focused on children, this training remains inconsistent and insufficient. Addressing these gaps will require a coordinated effort to develop and implement evidence-based, competency-aligned curricula that support the development of paediatricians who are equipped to promote ERH and resilience in children. Further research is needed to explore how such curricula can be mapped onto the Royal College competencies and integrated into Canadian paediatric programs, ultimately improving care for children and families.

## Supplementary Material

pxaf136_Supplementary_Data
