# Supplementary material for: Early relational health training in Canadian paediatric residency programs: A national program director survey
Source: Paediatr Child Health. 2026 Jan 23;31(3):200–6. doi: 10.1093/pch/pxaf136 (PMC13079103; doi:10.1093/pch/pxaf136)
Supplement: pxaf136_Supplementary_Data [file pxaf136_supplementary_data.zip › pch-see-25-005-File007.docx]

**Supplementary Material 2. General Pediatrics and Subspecialty Surveys (REB # 23/80X)**

**Early Relational Health in *General Pediatric* Training Programs in Canada**

Please feel free to copy this form and distribute to any affiliated teaching sites, if appropriate and applicable. Please mark “N/A” if a question is not applicable to your institution.

**Current context of your program:**

1. Please outline Early Relational Health is defined at your institution (e.g., 0-4 years, 0-5 years, 0-6 years)
2. With respect to **DIDACTIC TEACHING**:
   1. How many hours of lectures/teaching sessions are provided to residents?
   2. In what year or context are these provided? (e.g., Year 2 during core Mental Health rotation)
   3. What is the structure of this teaching? (e.g., lecture, small-group case-based)
3. With respect to **E-LEARNING** and other **SELF-DIRECTED LEARNING**:
   1. Do you have an e-learning module on Early Relational Health?
   2. Do you give residents a recommended reading list on Early Relational Health?
   3. Are these activities required or optional for residents?
      1. If optional, how many residents complete them?
4. With respect to **CLINICAL EXPERIENCES**:
   1. Are residents required to complete an Early Relational Health rotation or any experience in Early Relational Health?
      1. If yes, what is the context of the experience? (e.g., Early Relational Health Clinic, General Outpatient rotation with attention to ensuring residents are involved with a minimum number of young children)
      2. What is the length of the experience? (e.g., 3-month block rotation, longitudinal with a single family)
      3. Do any of the experiences include evidence-based interventions? (e.g., Parent-Child Interaction Therapy, Triple P, Circle of Security, etc.)
   2. Are elective opportunities in Early Relational Health available?
      1. If yes, how many residents participate in these elective each year?
5. With respect to **RESEARCH OPPORTUNITIES**:
   1. Are there any opportunities for residents to participate in Early Relational Health-related research projects?
6. How many staff affiliated with your program have an expressed interest or expertise in Early Relational Health?
   1. How many are involved in teaching activities?
   2. How many are involved in research?
7. How well do you think your program educates pediatric residents on parenting behaviours that promote children’s early (birth to 5 years) cognitive and socioemotional development (such as positive discipline, responding to cries, infant sleep, parent-child relationships, etc.)?

- Not very well
- Moderately well
- Very well
  - 1. If not very well, why? (check all that apply)
    - Cost
    - Time
    - Faculty buy-in
    - Competing priorities
    - No curriculum
    - Lack of faculty experience
    - Other (please specify): __________

1. Upon completion of your program, how knowledgeable do you your think residents are at the following?

|  | **Not knowledgeable** | **Somewhat knowledgeable** | **Knowledgeable** | **Very Knowledgeable** |
| --- | --- | --- | --- | --- |
| Understanding the effects of toxic stress on health outcomes. |  |  |  |  |
| Understanding the importance of caregiver-child attachment in social-emotional health. |  |  |  |  |
| Establishing a trusting relationship with caregivers. |  |  |  |  |
| Counselling caregivers on positive discipline. |  |  |  |  |
| Counseling caregivers on stimulating cognitive development. |  |  |  |  |
| Counseling caregivers on how to best support emotion regulation. |  |  |  |  |
| Counselling caregivers on how to respond to a very young child’s need. |  |  |  |  |
| Counseling caregivers on healthy sleep strategies. |  |  |  |  |

1. How important do you think a curriculum that addresses the above topics is in a residency training program?
   - Not important
   - Somewhat important
   - Important
   - Very important

**Curriculum Development in Early Relational Health**

A new curriculum has been designed by Dr. Blair Hammond et al., called the *“Keystones of Development”* that aims to in promoting parenting behaviours as a means to improve child development outcomes in visits with young children. Previous work evaluating the curriculum has shown to be effective in increasing resident behaviours, knowledge, and self-efficacy in this area. We are exploring interest in making it available to residency programs like yours throughout the country at no charge.

1. Which of the following would motivate you to implement a curriculum in your own residency program? Check all that apply
   - - Curriculum can be woven into the existing program without displacing any topics
     - Curriculum addresses specific core competencies of your program
     - Evidence that the quality of the caregiver-child relationship influences child outcomes
     - Evidence that the curriculum increases residents’ knowledge and skills
     - Evidence that this curriculum improves patient satisfaction
     - Evidence of residents’ satisfaction with the curriculum
     - Other (please specify): ___________
2. Which of the following free resources would you consider implementing at your program? Check all that apply
   - Self-guided learning modules (no faculty facilitation required) that model weaving caregiver curriculum into visits with young children and their caregiver(s)
   - Learning resources (videos, handouts, tips) for residents on speciﬁc parenting topics (e.g., discipline, attachment, routines, sleep strategies)
   - Learning resources (videos, handouts, tips) for caregivers on speciﬁc parenting topics (e.g., discipline, attachment, routines, sleep strategies)
   - Power Point slides for faculty lectures on speciﬁc parenting topics (e.g., discipline, attachment, routines, sleep strategies)
   - Parenting tips that can be emailed or texted to residents
   - Parenting tips that can be emailed or texted to caregivers
   - Live warmline support related to curriculum topics and additional parenting questions (for residents)
   - Other (please specify): __________

## What barriers would you face if you wanted to implement a free online curriculum at your program? Check all that apply.

## A lack of "buy-in" from attending physicians that this is a priority

## Residents won't have time to complete

## A lack of faculty with the requisite knowledge base to implement, oversee, or answer questions

## More important priorities to implement

## Other (please specify): ___________

1. What topics would you like covered? Check all that apply.
   - Positive discipline
   - Healthy sleep habits
   - Promoting secure caregiver-child attachment
   - Promoting emotion regulation
   - Tantrums
   - Promotion emotional literacy (e.g., communicating and expressing feelings)
   - Promoting independent exploration skills

**Your program**

## Are you a...

- General Pediatrician: private-practice/outpatient
- General Pediatrician: hospitalist / inpatient
- Subspecialist (please specify): ________
- Other (please specify): _______

## What is the size of your residency program?

- Small (<10 residents/year)
- Medium (10-30 residents/year)
- Large (>30 residents/year)

## What is the title of the person who completed most of this survey?

## Program Director

## Associate Program Director

## Continuity Clinic Director

## Chief Resident

## Other (please specify): ________

## Are you a preceptor in a residency clinic?

## Yes

## No

## Last year what percentage of residents at your program went into general outpatient pediatrics?

- <25%
- 25-49%
- 50-75%
- >75%

## In which setting do your residents see patients?

## Urban

## Suburban

## Rural

## All of the above

**Additional Comments:**

- - - 1. Please feel free to add any additional comments on Early Relational Health in your training program:

Thank you for your participation and feedback.

**Early Relational Health in *Pediatric Subspecialty Fellowship* Training Programs in Canada**

Please feel free to copy this form and distribute to any affiliated teaching sites, if appropriate and applicable. Please mark “N/A” if a question is not applicable to your institution.

**Current context of your program:**

1. Please outline Early Relational Health is defined at your institution (e.g., 0-4 years, 0-5 years, 0-6 years)
2. With respect to **DIDACTIC TEACHING**:
   1. How many hours of lectures/teaching sessions are provided to residents?
   2. In what year or context are these provided? (e.g., Year 2 during core Mental Health rotation)
   3. What is the structure of this teaching? (e.g., lecture, small-group case-based)
3. With respect to **E-LEARNING** and other **SELF-DIRECTED LEARNING**:
   1. Do you have an e-learning module on Early Relational Health?
   2. Do you give residents a recommended reading list on Early Relational Health?
   3. Are these activities required or optional for residents?
      1. If optional, how many residents complete them?
4. With respect to **CLINICAL EXPERIENCES**:
   1. Are residents required to complete an Early Relational Health rotation or any experience in Early Relational Health?
      1. If yes, what is the context of the experience? (e.g., Early Relational Health Clinic, General Outpatient rotation with attention to ensuring residents are involved with a minimum number of young children)
      2. What is the length of the experience? (e.g., 3-month block rotation, longitudinal with a single family)
      3. Do any of the experiences include evidence-based interventions? (e.g., Parent-Child Interaction Therapy, Triple P, Circle of Security, etc.)
   2. Are elective opportunities in Early Relational Health available?
      1. If yes, how many residents participate in these electives each year?
5. With respect to **RESEARCH OPPORTUNITIES**:
   1. Are there any opportunities for residents to participate in Early Relational Health-related research projects?
6. How many staff affiliated with your program have an expressed interest or expertise in Early Relational Health?
   1. How many are involved in teaching activities?
   2. How many are involved in research?
7. How well do you think your program educates pediatric residents on parenting behaviours that promote children’s early (birth to 5 years) cognitive and socioemotional development (such as positive discipline, responding to cries, infant sleep, parent-child relationships, etc.)?

- Not very well
- Moderately well
- Very well
  - 1. If not very well, why? (check all that apply)
    - Cost
    - Time
    - Faculty buy-in
    - Competing priorities
    - No curriculum
    - Lack of faculty experience
    - Other (please specify): __________

1. Upon completion of your program, how knowledgeable do you your think residents are at the following?

|  | **Not knowledgeable** | **Somewhat knowledgeable** | **Knowledgeable** | **Very Knowledgeable** |
| --- | --- | --- | --- | --- |
| Understanding the effects of toxic stress on health outcomes. |  |  |  |  |
| Understanding the importance of caregiver-child attachment in social-emotional health. |  |  |  |  |
| Establishing a trusting relationship with caregivers. |  |  |  |  |
| Counselling caregivers on positive discipline. |  |  |  |  |
| Counseling caregivers on stimulating cognitive development. |  |  |  |  |
| Counseling caregivers on how to best support emotion regulation. |  |  |  |  |
| Counselling caregivers on how to respond to a very young child’s need. |  |  |  |  |
| Counseling caregivers on healthy sleep strategies. |  |  |  |  |

1. How important do you think a curriculum that addresses the above topics is in a fellowship training program?
   - Not important
   - Somewhat important
   - Important
   - Very important

**Curriculum Development in Early Relational Health**

A new curriculum has been designed by Dr. Blair Hammond et al., called the *“Keystones of Development”* that aims to in promoting parenting behaviours as a means to improve child development outcomes in visits with young children. Previous work evaluating the curriculum has shown to be effective in increasing resident behaviours, knowledge, and self-efficacy in this area. We are exploring interest in making it available to fellowship programs like yours throughout the country at no charge.

- - - 1. Which of the following would motivate you to implement a curriculum in your own fellowship program? Check all that apply
    - Curriculum can be woven into the existing program without displacing any topics
    - Curriculum addresses specific core competencies of your program
    - Evidence that the quality of the caregiver-child relationship influences child outcomes
    - Evidence that the curriculum increases residents’ knowledge and skills
    - Evidence that this curriculum improves patient satisfaction
    - Evidence of residents’ satisfaction with the curriculum
    - Other (please specify): ___________
      1. Which of the following free resources would you consider implementing at your program? Check all that apply.
         - Self-guided learning modules (no faculty facilitation required) that model weaving caregiver curriculum into visits with young children and their caregiver(s)
         - Learning resources (videos, handouts, tips) for residents on speciﬁc parenting topics (e.g., discipline, attachment, routines, sleep strategies)
         - Learning resources (videos, handouts, tips) for caregivers on speciﬁc parenting topics (e.g., discipline, attachment, routines, sleep strategies)
         - Power Point slides for faculty lectures on speciﬁc parenting topics (e.g., discipline, attachment, routines, sleep strategies)
         - Parenting tips that can be emailed or texted to residents
         - Parenting tips that can be emailed or texted to caregivers
  - Live warmline support related to curriculum topics and additional parenting questions (for residents)
  - Other (please specify): __________
    - 1. What barriers would you face if you wanted to implement a free online curriculum at your program? Check all that apply.

## A lack of "buy-in" from attending physicians that this is a priority

## Residents won't have time to complete

## A lack of faculty with the requisite knowledge base to implement, oversee, or answer questions

## More important priorities to implement

## Other (please specify): ___________

- - - 1. What topics would you like covered? Check all that apply.
  - Positive discipline
  - Healthy sleep habits
  - Promoting secure caregiver-child attachment
  - Promoting emotion regulation
  - Tantrums
  - Promotion emotional literacy (e.g., communicating and expressing feelings)
  - Promoting independent exploration skills

**Your program**

## Are you a...

- General Pediatrician: private-practice/outpatient
- General Pediatrician: hospitalist / inpatient
- Subspecialist (please specify): ________
- Other (please specify): _______

## What is the size of your fellowship program?

- Small (<5 residents/year)
- Medium (5-10 residents/year)
- Large (>10 residents/year)

## What is the title of the person who completed most of this survey?

## Program Director

## Associate Program Director

## Continuity Clinic Director

## Chief Resident

## Other (please specify): ________

## Are you a preceptor in a residency/fellowship clinic?

## Yes

## No

## Last year what percentage of residents at your program went into general outpatient pediatrics?

- <25%
- 25-49%
- 50-75%
- >75%

## In which setting do your residents see patients?

## Urban

## Suburban

## Rural

## All of the above

**Additional Comments:**

- - - 1. Please feel free to add any additional comments on Early Relational Health in your training program:

Thank you for your participation and feedback.
